# Cucumber (*Cucumis sativus* L.) Seedling Rhizosphere *Trichoderma* and *Fusarium* spp. Communities Altered by Vanillic Acid

**DOI:** 10.3389/fmicb.2018.02195

**Published:** 2018-09-18

**Authors:** Shaocan Chen, Hongjie Yu, Xingang Zhou, Fengzhi Wu

**Affiliations:** ^1^Department of Horticulture, Northeast Agricultural University, Harbin, China; ^2^Key Laboratory of Biology and Genetic Improvement of Horticultural Crops (Northeast Region), Ministry of Agriculture, Harbin, China

**Keywords:** *Trichoderma* spp., *Fusarium* spp., fungal community, *Cucumis sativus* L., vanillic acid

## Abstract

Root exudates mediate soil microbiome composition and diversity, which might further influence plant development and health. Vanillic acid from root exudates is usually referred as autotoxin of cucumber, however, how vanillic acid affect soil microbial community diversities and abundances remains unclear. In this study, vanillic acid (VA; 0.02, 0.05, 0.1, and 0.2 μmol g^-1^ soil) was applied to soil every other day for a total of five applications. We used Illumina MiSeq sequencing, quantitative PCR (qPCR) and PCR-denaturing gradient gel electrophoresis (PCR-DGGE) to test the effects of VA on the total fungi community composition as well as the *Trichoderma* and *Fusarium* spp. community abundances and structures in the cucumber rhizosphere. Illumina MiSeq sequencing showed that VA (0.05 μmol g^-1^ soil) increased the relative abundance of the fungal phylum Basidiomycota while decreasing the relative abundance of Ascomycota (*P* < 0.05), and not altered the diversity of the soil fungal community. VA (0.05 μmol g^-1^ soil) also increased the relative abundances of the fungal genera with plant pathogens, such as *Conocybe* and *Spizellomyces* spp.(*P* < 0.05). A qPCR analysis showed that VA (0.05 to 0.2 μmol g^-1^ soil) exerted promoting effects on *Trichoderma* spp. community abundance and stimulated *Fusarium* spp. abundance at low concentrations (0.02 to 0.05 μmol g^-1^ soil) but inhibited it at high concentrations (0.1 to 0.2 μmol g^-1^ soil). The PCR-DGGE analysis showed that all concentrations of VA altered the community structures of *Trichoderma* spp. and that the application of VA (0.02 and 0.05 μmol g^-1^ soil) changed the band number and the Shannon-Wiener index of the *Fusarium* spp. community. This study demonstrated that VA changed the total fungal community in the cucumber seedling rhizosphere and that the *Trichoderma* and *Fusarium* spp. communities showed different responses to VA.

## Introduction

Autotoxicity is an intraspecific allelopathy process through which plants can inhibit their growth or that of their relatives by releasing toxic chemicals into the environment (Huang et al., 2013), as has been observed in both natural and manipulated ecosystems, particularly agroecosystems. According to previous studies, an imbalance in the microbial community structure and the accumulation of soil-borne pathogens induced by autotoxins are the main causes of soil sickness (Yu et al., 2000; Lin et al., 2015; Zhang et al., 2016). Cucumber is one of the main vegetable crops among greenhouse plants and is planted in up 40% of the greenhouse vegetable-producing area in China; however, soil sickness problems substantially restrict cucumber production (Yu et al., 2000). Phenolic acids (including derivatives of cinnamic and benzoic acids), which have harmful effects on cucumber performance, are potential autotoxins of cucumber in both hydroponic and soil culture (Blum, 1996; Zhou et al., 2017).

Soil microorganisms play an important role in sustaining terrestrial ecosystem processes, and the soil microbial community abundance and diversity are sensitive to fertilization, irrigation, and plant history (Mendes et al., 2011; Bardgett and van der Putten, 2014; Zhou et al., 2017). Previous studies shown that phenolic acid significantly decreases the functional and genetic diversity of the soil microbial community (Wu et al., 2008a) and selectively increases the species of certain microorganisms (Qu and Wang, 2008). Recent studies involving *in vitro* experiments or soil experiments have stressed the effects of phenolic acids, such as ferulic (Zhou and Wu, 2012b), vanillin (Zhou et al., 2018a) and *p*-coumaric acid (Zhou et al., 2018b), on specific microorganisms or soil microbial communities. Moreover, several soil microorganisms can use phenolic compounds as specific substrates or signaling molecules (Badri et al., 2013). Thus, phenolic acid might also affect the activity and diversity of soil microbial communities (Zhou et al., 2012; Gu et al., 2016). However, many phenolic acids have also been identified as autotoxins from plants, root exudates, and soil (Huang et al., 2013), and the effects of these acids on the microbial communities in soil remains unclear.

*Fusarium* spp. contain many phytopathogenic species, which can infect a wide range of crop plants and cause vascular wilt disease (Ma et al., 2013). *Trichoderma* spp. are opportunistic, act as biocontrol agents and have the ability to promote plant growth and development (Harman, 2006). For example, *T. asperellum* (T-34) and *Trichoderma harzianum* (SQR-T307) are efficient biological control agents against *Fusarium oxysporum* (Cotxarrera et al., 2002; Yang et al., 2011), and *T. asperellum* (T-203), and *T. harzianum* (Tr6) can induce systemic resistance in cucumber (Yedidia et al., 2003; Alizadeh et al., 2013). *In vitro* studies have demonstrated that phenolic acids can affect the growth and physiological status of specific microorganisms from *Fusarium* and *Trichoderma* spp. communities (Corrales et al., 2010; Lanoue et al., 2010). In addition to crops under continuous monocropping (Okoth et al., 2007; Zhou and Wu, 2012a), phenolic acid could also affect the soil *Fusarium* and *Trichoderma* spp. Communities (Zhou et al., 2018a). However, accumulating evidence indicates that different phenolic acids exert different effects on the same soil microorganisms (Liu et al., 2016; Zhou and Wu, 2018; Zhou et al., 2018b).

Vanillic acid is a common phenolic acid detected in cucumber root exudates (Pramanik et al., 2000), and its concentration in cucumber rhizosphere under continuous monocropping is approximately 0.05 μmol g^-1^ soil (Zhou et al., 2012). We hypothesize that vanillic acid might influence the composition of the fungal community because many soil microorganisms can use phenolic compounds as carbon resources. According to recent reports, the accumulated autotoxins in rhizosphere soil increase the number of harmful microorganisms and decrease the number of beneficial microorganisms (Huang et al., 2013; Lin et al., 2015). We further hypothesized that vanillic acid might stimulate the *Fusarium* spp. community and suppress the *Trichoderma* spp. community in soil. The objectives of this study were (1) to evaluate the effects of vanillic acid on the soil fungal community through the high-throughput sequencing of the internal transcribed spacer 1 (ITS1) gene and (2) to detect the responses of the *Trichoderma* and *Fusarium* spp. communities to vanillic acid through quantitative PCR (qPCR) and PCR-denaturing gradient gel electrophoresis (PCR-DGGE) analyses.

## Materials and Methods

### Greenhouse Experiment

Soil samples were collected from an open field at the Northeast Agricultural University Experimental Station in Harbin, China (lat. 45°41′ N, long. 126°37′ E) that was covered with grass and had been undisturbed for more than 15 years. Ten soil cores were sampled from the surface layer (0–15 cm), thoroughly mixed, and sieved (4 mm) prior to use. The soil was a black soil (Chernozem) composed of 326 g kg^-1^ sand, 384 g kg^-1^ silt, 270 g kg^-1^ clay, 36.7 g kg^-1^ organic matter, 89.0 mg kg^-1^ available nitrogen, 63.4 mg kg^-1^ available phosphorus and 119 mg kg^-1^ available potassium, with an EC (1:2.5, w/v) of 0.33 mS cm^-1^ and a pH (1:2.5, w/v) of 7.78.

A pot experiment was conducted in a greenhouse (day and night temperatures of 32 and 22°C, respectively, relative humidity of 60–80%, 16-h light/8-h dark cycle) to test the effects of VA on the total fungal, *Trichoderma* and *Fusarium* spp. communities in the cucumber rhizosphere. As described by Shafer and Blum (1991), we applied VA to the soil periodically to maintain the desired level because soil microorganisms degrade phenolic acids under favorable environmental conditions. We transplanted cucumbers (cv. Jinlv 3) with two cotyledons into pots that were filled with 150 g of soil. After the cucumber plants reached the one-leaf stage, we then added different concentrations of VA (0.02, 0.05, 0.1, and 0.2 μmol g^-1^ soil) every 2 days for a total of five applications as described by Zhou and Wu (2013). To eliminate the influence of soil pH, 0.1 M NaOH was used to adjust the pH of the solution to 7.0. Soil treated with distilled water was used as a control (0). The soil water content was adjusted every 2 days with distilled water to maintain constant pot weights. The experiment comprised five treatments: four concentrations of VA and the control treatment. Each treatment was applied to five pots, and the experiment was replicated three times. Each pot contained one cucumber seedling.

### Rhizosphere Soil Sampling

As described by Zhou and Wu (2013), 10 days after the first application of vanillic acid, the cucumber roots were removed from the pot, and the cucumber seedling was manually shaken to remove the soil that was loosely attached to cucumber roots. The soil that was tightly adhered to roots was considered rhizosphere soil and removed from the root surface using a sterile brush. Cucumber rhizosphere soil samples were collected from five plants of each treatment replicate and mixed to obtain a composite sample, and after sieving (2 mm), the fresh soil samples were immediately stored at -70°C for DNA extraction. Three composite samples were obtained for each treatment.

Rhizosphere soil (250 mg) DNA was extracted with a PowerSoil DNA Isolation Kit (MO BIO Laboratories, Carlsbad, CA, United States) according to the manufacturer’s instructions. Three DNA extractions were performed using each composite soil sample, and the extracted DNA solutions were pooled.

### Illumina MiSeq Sequencing of Fungal Communities

Zhou et al. (2012) previously found that in a continuous monocropping system, the concentration of vanillic acid in the cucumber rhizosphere was approximately 0.05 μmol g^-1^ soil. Thus, we performed an assessment of fungal communities through Illumina MiSeq sequencing of the total soil DNA obtained from the 0 and 0.05 μmol g^-1^ soil VA treatments. The PCR assay was performed in triplicate in a 20-μL mixture containing 2 μL of 10× Fast Pfu buffer, 2 μL of 2.5 mM dNTPs, 0.8 μL of each primer (5 μM), 0.4 μL of Fast Pfu polymerase, 10 ng of template DNA, and a sufficient volume of ddH_2_O to obtain a final volume of 20 μL. The ITS1 regions of the fungal rRNA genes were amplified by PCR using the primers ITS1F/ITS2 as follows: 95°C for 3 min, 35 cycles at 94°C for 30 s, 55°C for 30 s, and 72°C for 45 s, and a final extension at 72°C for 10 min. The PCR amplifications were purified using an agarose gel DNA purification kit (TaKaRa). The PCR products were detected and quantified using a QuantiFluor^TM^-ST blue fluorescence quantitative system (Promega Corporation) and then sequenced using the amount of DNA that allowed each sample to be mixed with the appropriate proportions. The mixtures were then pyrosequenced using a MiSeq Genome Sequencer PE300 Titanium platform (Majorbio Bio-Pharm Technology, Co., Ltd., Shanghai, China).

Raw FASTQ files were de-multiplexed, quality-filtered and processed using QIIME (version 1.17) (Caporaso et al., 2010), and raw sequences were merged using FLASH (fast length adjustment of short reads) software (Magoć and Salzberg, 2011). Sequences with >1 N (ambiguous bases) in the reads and expected errors > 0.5 were discarded. The molecular identifier tags and primer sequences were removed allowing 0 and 2 mismatches, respectively. The UPARSE software package was applied for de-noising and chimera detection (Edgar, 2013). Sequences were binned to operational taxonomic units (OTUs) at 97% sequence similarity with UPARSE using an agglomerative clustering algorithm (Edgar, 2013). Taxonomic assignments for the OTUs were performed through a BLAST search against the Unite database (Kõljalg et al., 2013). All the identified sequences were deposited into the NCBI-Sequence Read Archive and mentioned in **Supplementary Table S1**.

### PCR-DGGE Analysis

The soil *Trichoderma* and *Fusarium* spp. community structures were estimated using the PCR-DGGE method. Partial *Trichoderma* spp. ITS regions and the *Fusarium* spp. *Ef1α* gene were nest-amplified. The primer sets ITS1F/ITS4*Tr*R and ITS*Tr*F-GC/ITS*Tr*R were used as described by Meincke et al. (2010) for amplification of the *Trichoderma* ITS regions and EF-1/EF-2 (O’Donnell et al., 1998) and Alfie1-GC/Alfie2 (Yergeau et al., 2005) were used for amplification of the *Fusarium* spp. *Ef1α* gene in the first and second PCR amplification rounds, respectively.

Denaturing gradient gel electrophoresis analysis of the *Trichoderma* spp. community was performed using a 6–9% (w/v) acrylamide gel with a 30–60% denaturation gradient, and the *Fusarium* spp. community was analyzed using a 6% (w/v) acrylamide gel with a 40–60% denaturation gradient. All the gels were run in 1× TAE (Tris acetate-EDTA) buffer for 12 h at 60°C and 80 V with a DCode universal mutation detection system (Bio-Rad Lab, Christiansburg, VA, United States). After electrophoresis, the gels were stained in a 1:3300 (v/v) GelRed (Biotium, Fremont, CA, United States) nucleic acid staining solution for 20 min. The DGGE profiles were photographed with an AlphaImager HP imaging system (Alpha Innotech, Corp., San Leandro, CA, United States) under 302-nm UV light.

### qPCR Assay

The abundances of the soil *Trichoderma* and *Fusarium* spp. communities were estimated through real-time PCR assays with an IQ5 Real-time PCR system (Bio-Rad Lab, LA, United States). The *Trichoderma* spp. community was quantified with UtF/UtR as described by Hagn et al. (2007). The *Fusarium* spp. community was quantified by nest amplification of the *Ef1α* gene with EF-1/EF-2 (O’Donnell et al., 1998) and Alfie1/Alfie2 (Yergeau et al., 2005). The first-round amplification of the *Ef1α* gene was conducted using an S1000^TM^ Thermal Cycler (Bio-Rad Lab, LA, United States) in a 50-μL reaction mixture that contained 25 μL of 2× Taq PCR MasterMix (Tiangen Biotech, Beijing, China), 0.2 μM of each primer and 5 ng of soil DNA. The PCR conditions were as follows: (1) 94°C for 5 min, (2) 32 cycles of 94°C for 45 s, 55.5°C for 45 s, and 72°C for 90 s for the *Trichoderma* spp. ITS region or 35 and 30 cycles of 94°C for 45 s, 53°C and 67°C for 45 s and 72°C for 90 s for the first- and second-round amplification of the *Fusarium Ef1α* gene, respectively, and (3) 72°C for 10 min. Standard curves were created with 10-fold dilution series of plasmids containing the ITS regions from soil samples, and these curves were then used for the analysis of *Trichoderma* spp. communities. The initial copy number of the target gene was calculated by comparing the threshold cycle (Ct) values of each sample with the standard curve. The relative *Fusarium* spp. community abundance was calculated as described by Stevena et al. (2008). Sterile water was used as a negative control.

All amplifications were conducted three times. The specificity of the products was confirmed by melting curve analysis and agarose gel electrophoresis. The CT values obtained for each sample were compared with the standard curve to determine the initial copy number of the target gene.

### Statistical Analysis

The Shannon and inverse Simpson diversity indices for the Illumina MiSeq sequencing data were calculated using QIIME (Caporaso et al., 2010). For the analysis of beta diversity, the microbial community composition (relative OTU abundance data) was investigated through a principal coordinate analysis (PCoA) based on a Bray–Curtis distance matrix, and the coordinates were used to draw 2D graphical outputs. ANOSIM, adonis, and MRPP analyses were performed to test the differences in microbial communities using the Bray–Curtis distance and 999 permutations. The ANOSIM, adonis, and MRPP results were processed with the “vegan” package in “R” (Team 2013).

For the PCR-DGGE data, the banding patterns of the DGGE profiles and PCA analysis were performed using Quantity One software (version 4.5) and Canoco for Windows software (version 4.5) (Matsuyama et al., 2007), respectively. The density value of each band was divided by the average band density of the lane to minimize the influence of the loaded DNA concentrations among samples (Graham and Haynes, 2005). The number of bands and Shannon-Wiener index of the *Trichoderma* and *Fusarium* communities structures were calculated as described previously (Liu et al., 2007).

The alpha diversity indices and relative abundances of fungal taxa were analyzed by analysis of variance (ANOVA). The means of the alpha diversity indices and relative abundances of fungal taxa were compared between treatments using Welch’s *t*-test. The data from the DGGE analysis and the soil microbial abundances determined from the qPCR assay were analyzed by ANOVA, and the means of the different treatments were compared based on Tukey’s honestly significant difference (HSD) test. The differences were considered statistically significant if *P* < 0.05.

## Results

### Sequence Summary by High-Throughput Amplicon Sequencing

A total of six samples were obtained from the various treatments (0 and 0.05). To compare the taxonomic diversity among the six samples, we normalized the sequence number of each sample to 32,073 reads (the lowest number of reads among the six samples). The number of OTUs and Shannon-Wiener curves indicated that the sequencing data represented most of the total fungal community composition (**Supplementary Figure S1**).

### Vanillic Acid Alters Fungal Community Composition

A phylum-level analysis of the communities revealed that 2.03% of the fungal sequences were unclassified, and all the samples were dominated (>90%) by only two phyla, namely, Ascomycota and Zygomycota. The relative abundance of Ascomycota was lower and the relative abundances of Fungi unclassified was higher in vanillic acid-treated soil (0.05) compared with the control soil (*P* < 0.05) (**Supplementary Figure S2A**).

**FIGURE 1:**
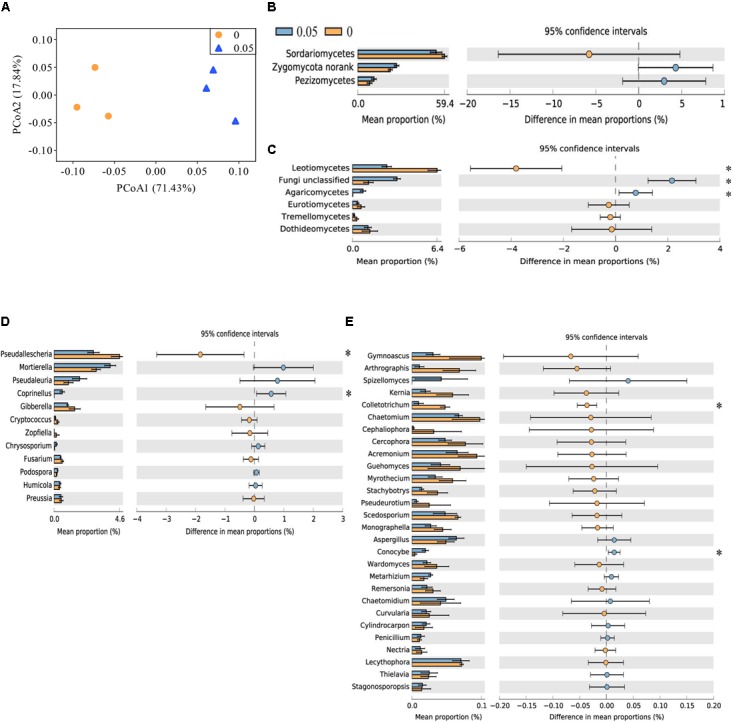
Principal coordinate analysis (PCoA) analysis of soil fungal community and taxonomic characteristic of fungal communities in cucumber rhizosphere treated with water (0) and vanillic acid (0.05 μmol g^-1^ soil). The PCoA plot **(A)** was based on the Bray–Curtis distance at the OTU level (97% sequence similarity) of fungal community. Relative abundances of major soil fungal classes **(B,C)** and major classified fungal genera **(D,E)** in the vanillic acid (0.05 μmol g^-1^ soil) and water-treated soils (0). Fungal classes with average relative abundances > 7% **(B)** and > 0.1% **(C)** were shown in at least one treatment. Fungal genera with average relative abundances > 0.1% **(D)** and > 0.01% **(E)** were shown in at least one treatment. Values in the bar plot are expressed as mean ± standard error. The colored circles represent the 95% confidence intervals. Asterisks indicate significant difference between treatments based on Welch’s *t*-test (*P* < 0.05).

The top three classes were Sordariomycetes, Zygomycota norank and Pezizomycetes, which accounted for approximately 91% of the fungal sequences. Compared with the control treatment, the soil treated with 0.05 μmol g^-1^ soil VA had higher relative abundances of Agricomycetes and Fungi unclassified but a lower relative abundance of Leotiomycetes (*P* < 0.05) (**Figures 1B,C**).

At the order level, Sordariales and Mortierellales were the dominant orders (average relative abundance > 20%) in all the samples. The relative abundances of Microascales and Thelebolales were lower and those of Fungi unclassified, Agaricales and Auriculariales were higher in vanillic acid-treated soil (0.05) compared with the control soil (*P* < 0.05) (**Supplementary Figures S2B,C**).

At the family level, Chaetomiaceae, Mortierellaceae, and Lasiosphaeriaceae were the dominant families (average relative abundance > 10%) in all the samples. The relative abundances of Pezizales unclassified, Fungi unclassified, Psathyrellaceae, Auriculariales unclassified and Bionectriaceae were higher and those of Lasiosphaeriaceae and Microascaceae were lower in vanillic acid-treated soil (0.05) compared with control soil (*P* < 0.05) (**Supplementary Figures S2D,E**).

More than 100 fungal genera were detected across all the samples analyzed (data not shown). The relative abundances of *Coprinellus* and *Conocybe* spp. were higher and those of *Pseudallescheria* and *Colletotrichum* spp. were lower in vanillic acid-treated soil (0.05) than in control soil (*P* < 0.05) (**Figures 1D,E**).

**FIGURE 2:**
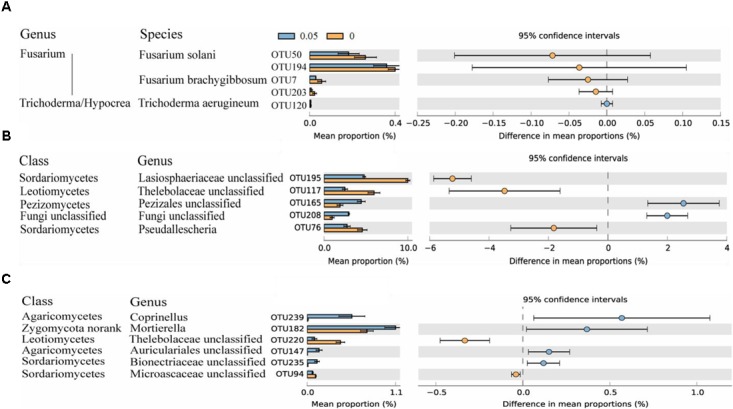
Number of sequences belonging to *Fusarium* and *Trichoderma*/*Hypocrea* spp. as determined by Illumina MiSeq sequencing and relative abundances of significantly altered fungal OTUs. Fungal OTUs belonging to *Fusarium* and *Trichoderma*/*Hypocrea* spp. were chose **(A)**. Fungal OTUs with average relative abundances > 0.5% **(B)** and > 0.1% **(C)** were shown in at least one treatment. Values in the bar plot are expressed as mean ± standard error. The colored circles represent the 95% confidence intervals.

A total of 249 OTUs at 97% similarity were identified among the six samples. Most dominant OTUs (relative abundance > 0.1%) were aligned with the fungal phyla Ascomycota and Zygomycota. Soil treated with 0.05 μmol g^-1^ soil VA showed higher relative abundances of six OTUs that aligned with Pezizales unclassified, Fungi unclassified, Auriculariales unclassified, Bionectriaceae unclassified, *Mortierella*, and *Coprinellus* spp. The control soil was enriched with five dominant OTUs that aligned with Lasiosphaeriaceae unclassified, Thelebolaceae unclassified, Microascaceae unclassified and *Pseudallescheria* spp. (*P* < 0.05) (**Figures 2B,C**).

### Vanillic Acid Does Not Alter Alpha and Beta Diversities of Fungal Community

The number of fungal OTUs, Shannon and inverse Simpson index were not significantly differ between vanillic acid-treated soils and control soils (**Supplementary Figure S3**). Principal coordinates analysis, based on Bray–Curtis distance matrix revealed that the fungal communities in the vanillic acid-treated soil (0.05) were distinctly different from that of the control soil (**Figure 1A**), but the difference was not statistically significantly (ANOSIM, *R* = 1, *P* = 0.100; adonis, *F* = 8.875, *R*^2^ = 0.689, *P* = 0.100; MRPP, delta = 0.086, effect size = 0.357, *P* = 0.100).

### Vanillic Acid Does Not Alter the *Trichoderma* and *Fusarium* Community Compositions

Through MiSeq sequencing, four OTUs were classified as *Fusarium* spp., and one OTU was classified as *Trichoderma*/*Hypocrea* spp. Two OTUs of *Fusarium* spp. could not be aligned at the species level, and OTU50 and OTU7 were classified as *Fusarium solani* and *Fusarium brachygibbosum*, respectively. For *Trichoderma*/*Hypocrea* spp., OTU120 was classified as *Trichoderma aerugineum*. These five OTUs were detected in all the samples, and the similar sequence numbers were observed in the 0 and 0.05 treatments (**Figure 2A**).

### Vanillic Acid Alters the *Trichoderma* and *Fusarium* spp. Community Abundances

**FIGURE 3:**
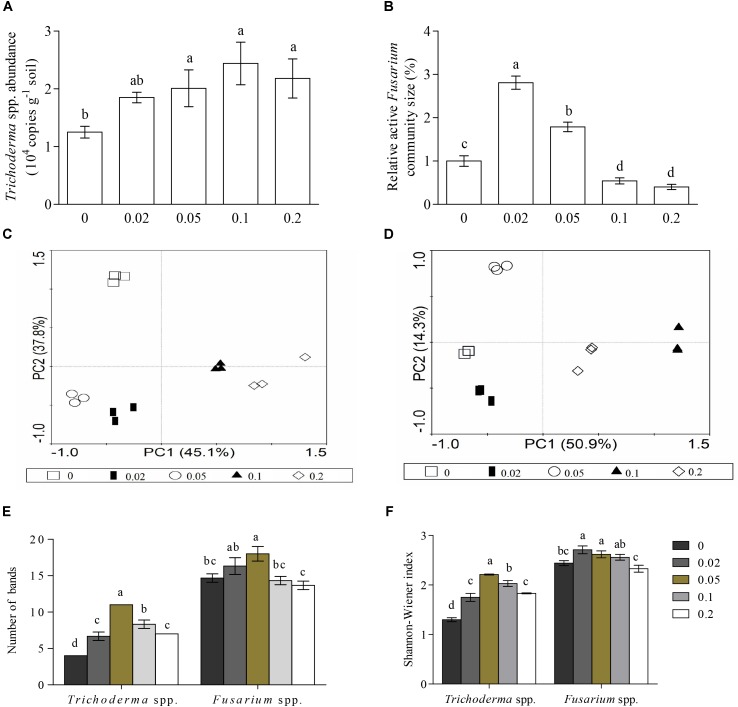
Abundances and diversities of *Trichoderma* and *Fusarium* spp. based on qPCR and PCR-DGGE analysis. **(A,B)** Represent *Trichoderma* and *Fusarium* spp. abundances, respectively. **(C,D)** Represent PCA analysis of *Trichoderma* and *Fusarium* spp., respectively. **(E,F)** Represent number of visible bands and Shannon-Wiener index, respectively. 0.02, 0.05, 0.1, and 0.2 indicate cucumber rhizosphere soils treated with vanillic acid at concentrations of 0.02, 0.05, 0.1, and 0.2 μmol g^-1^ soil, respectively. 0 represents samples treated with water. Different letters indicate significant differences between treatments (*P* < 0.05, Tukey’s HSD test).

The qPCR analysis showed that treatment with 0.05, 0.1, and 0.2 μmol g^-1^ soil VA significantly increased the soil *Trichoderma* spp. abundance (**Figure 3A**), and low concentrations of VA (0.02 to 0.05 μmol) increased the *Fusarium* spp. abundance, but high concentrations of VA (0.1 to 0.2 μmol) had the opposite effect (**Figure 3B**).

### Vanillic Acid Alters the *Trichoderma* and *Fusarium* spp. Community Structures

Visual inspection of the DGGE profiles showed that the DGGE banding patterns of the *Trichoderma* (**Supplementary Figure S4A**) and *Fusarium* spp. (**Supplementary Figure S4B**) communities were broadly similar in triplicate samples from each treatment. A PCA clearly distinguished the five treatments from each other (**Figures 3C,D**).

The number of visible bands and the Shannon-Wiener and Evenness indexes of the *Trichoderma* spp. community were significantly higher in the vanillic acid-treated soil compared with the control soil (*P* < 0.05), and these three indexes decreased with increases in the concentration of vanillic acid from 0.05 to 0.2 μmol g^-1^ soil. Compared with water-treated soil, both 0.02 and 0.05 μmol g^-1^ soil VA significantly increased the Shannon-Wiener index of the *Fusarium* spp. community, 0.02 μmol g^-1^ soil VA increased the Evenness index, and 0.05 μmol g^-1^ soil VA increased the number of visible bands of the *Trichoderma* spp. community (*P* < 0.05) (**Figures 3E,F**).

## Discussion

Soil microorganisms play an important role in the regulation of soil fertility and are closely correlated with the aboveground plant performance (Jousset et al., 2016). In this study, we focused on the effects of phenolic compounds on soil microbial communities, and the results revealed that vanillic acid had certain effects on the rhizosphere fungal community composition, which is consistent with previous observations of the peanut rhizosphere after treatment with benzoic acid (Liu et al., 2016). VA altered the community structure of *Trichoderma* spp. by increasing the number of bands and the Shannon-Wiener and evenness indices in the DGGE profile, and the tested concentrations (0.02–0.2 μmol g^-1^ soil) of VA increased the *Trichoderma* spp. abundance. In addition, low concentrations (0.02 and 0.05 μmol g^-1^ soil) of VA increased the abundance and diversity of *Fusarium* spp., whereas high concentrations (0.1 and 0.2 μmol g^-1^ soil) of VA decreased the abundance of *Fusarium* spp., indicating that our second hypothesis was not fully correct.

Many soil microorganisms can degrade phenolic compounds (Margesin et al., 2004). In this study, the relative abundances of several fungal taxa with phenol-degrading capability, including *Mortierella* (Inderjit, 2005), *Humicola* (Dao, 1987) and *Coprinellus* (Guiraud et al., 1999), were increased in the vanillic acid-treated soils, as demonstrated by MiSeq sequencing. These results confirmed previous findings that showed that phenolic compounds can stimulate phenolic acid-utilizing microorganisms in the rhizosphere (Blum et al., 2000). Future *in vitro* studies should focus on validating the capabilities of these microorganisms to metabolize VA.

The interactions between plant and soil microorganisms greatly determine the health and fitness of a plant (Chen et al., 2014; Li et al., 2014b). Huang et al. (2013) found that autotoxins influence soil microbes, leading to an increase in the degree of soil sickness. Inderjit et al. (2009) reported that the phytotoxic effects of catechin might be exerted through microbes in some soils. In this study, VA increased the relative abundances of fungal taxa that contain plant pathogens, such as *Conocybe* (Hallen et al., 2003) and *Spizellomyces* spp. (Lozupone and Klein, 2002), and inhibited plant–pathogen antagonism, such as *Pseudallescheria* spp. (Xu et al., 2012). We previously found that VA at a concentration ≥ 0.05 μmol g^-1^ soil inhibited cucumber seedling growth (Zhou and Wu, 2013). Thus, VA serves as an autotoxin that can affect cucumber growth by enhancing the growth of phytopathogenic fungi and inhibiting the growth of plant-beneficial fungi, and a plant-soil feedback experiment should be performed to evaluate this hypothesis.

In this study, qPCR and PCR-DGGE analyses showed that low concentrations (0.02 and 0.05 μmol g^-1^ soil) of VA increased the abundance and diversity of *Fusarium* spp., which indicated that VA selectively increases the species in the *Fusarium* spp. community. However, under the same experimental conditions, our previous study found that the *Fusarium* spp. abundance and diversity increases with increases in the VA concentration from 0.02 to 0.2 μmol g^-1^ soil (Zhou et al., 2018a). This finding might be attributed to the different effects of different phenolic compounds on the same genus. For example, benzoic acid affects the relative abundance of *Burkholderia* spp. (Liu et al., 2016), but VA does not affect these species (Zhou and Wu, 2018). In China, cucumber Fusarium wilt, which is considered the primary cause of poor cucumber production observed under greenhouse conditions with continuous monocropping, is caused by several *Fusarium* species, including *F. oxysporum* (the major pathogen), *F. equiseti*, *F. solani*, *F. moniliforme*, and *F. proliferatum*. Therefore, the significant increase in *Fusarium* abundance observed in response to VA treatment indicates that the presence of soilborne pathogens induced by some components of cucumber root exudates might contribute to the increased incidence of soilborne disease in cucumber monoculture soil. This hypothesis is supported by previous *in vitro* studies, which showed that VA stimulates *F. oxysporum f.* sp. *niveum* growth (Wu et al., 2008b) and that cinnamic acid promotes the incidence of Fusarium wilt in cucumber (Ye et al., 2004).

In contrast to the effects of low VA concentrations, high concentrations of VA (0.1 and 0.2 μmol g^-1^ soil) decreased the *Fusarium* spp. community abundance. An *in vitro* experiment showed that the growth of *Fusarium* species is inhibited by the presence of high VA concentrations (Samapundo et al., 2007; Zhao et al., 2018). Previous studies usually applied high concentrations of active phenolic acids that might not be relevant to the actual field conditions (Inderjit and Bhowmik, 2004; Inderjit et al., 2005). The concentrations of soil phenolic acids range from 0.01 to 0.5 μmol g^-1^ soil in natural and agricultural ecosystems (Muscolo and Sidari, 2006; Piotrowski et al., 2008; Li et al., 2018). The concentrations of VA used in this study was based on that reported by Zhou et al. (2012), who found that the concentration of VA in the cucumber rhizosphere in a continuous monocropping system is approximately 0.05 μmol g^-1^ soil. According to previous studies, the concentrations of VA used in this study (0.02 to 0.2 μmol g^-1^ soil) were within the realistic range of concentrations found in soil. However, the concentration of VA in cucumber continuous monocropping systems might not reach the abovementioned concentrations (0.1 and 0.2 μmol g^-1^ soil). Future studies should estimate the degradation of VA by monitoring the VA retained in soil.

Previous research showed that some species of *Trichoderma* spp. are able to degrade phenolic compounds (Derito and Madsen, 2008). In this study, compared with water-treated soil, VA increased the *Trichoderma* spp. abundance, the number of bands, and the Shannon-Wiener and Evenness indices, which was consistent with the results reported by Zhou et al. (2018a). However, increases in the concentration of VA from 0.05 to 0.2 μmol g*^-^*^1^ soil decreased the diversity of the genus but did not change the abundances. These results indicate that VA can stimulate or inhibit specific *Trichoderma* spp. groups in soil. *Trichoderma* spp. are starting to be used in reasonably large quantities in plant agriculture for both disease control and yield increases (Harman, 2006). For example, *T. harzianum* SQR-T037 can decompose phenolic compounds released by cucumber roots, thereby relieving the autotoxicity of cucumber (Chen et al., 2011). *Trichoderma atroviride* TRS25 can induce the resistance of cucumber against *Rhizoctonia solani* (Szczech et al., 2017). Thus, some taxa of *Trichoderma* spp. that were found to be stimulated by VA in this study might not be biocontrol agents or plant growth promoters, and this question should be studied in the future.

*Fusarium* and *Trichoderma* spp. showed very different responses to VA, and these responses largely depended on the species. Both *Fusarium* and *Trichoderma* spp. are filamentous fungi, which are the main fungus responsible for the degradation of phenolic compounds. Mason and Wasserman (1987) proposed mechanisms for the antimicrobial activity of phenolic compounds, and these mechanisms include enzyme inhibition by the oxidized compounds, possibly through reaction with a sulfhydryl group or through more non-specific interactions with proteins. Laccase from ascomycete, basidiomycete, and deuteromycete fungi have been implicated in lignin degradation and in protection against toxic phenolic monomers of polyphenols (Assavanig et al., 1992). Both *Fusarium* and *Trichoderma* species contain laccase compounds, but the effects of pH on the activities of these laccases are different. For example, the highest activity of the laccase in *F. solani* MAS2 was detected at pH 3.0 (Wu et al., 2010), whereas the maximal activity of the laccase of *T. harzianum* WL1 was observed at pH 4.5 (Sadhasivam et al., 2008). Other enzymes in *Fusarium* and *Trichoderma* spp. are involved in phenolic acid degradation (Cai et al., 2007). In this study, the *Trichoderma* spp. community abundance and diversity showed a positive response to VA amendment. Thus, our results for *Fusarium* and *Trichoderma* spp. indicated that the net effects of phenolic acid on the soil fungal community cannot be simply summarized as positive or negative.

In continuous monocropping systems, crop roots repeatedly release the same types of exudates for many years, which occasionally results in significant colonization and infection by certain beneficial or pathogenic microorganisms that utilize these substrates (Li et al., 2014a). In general, the rhizosphere microbial communities have higher abundances but lower diversities than those of the bulk soil (Berendsen et al., 2012). Previous studies have shown that exogenous phenolic acid can also change the composition and abundance of soil microbial communities in the absence of host plants. Yu and Matsui (Jing and Matsui, 1997) found that increases in ion leakage might be attributed to phenolic compound-induced damage to plant roots and that these changes in root exudates might also alter the rhizosphere microbial communities. Thus, VA might also change the rhizosphere microbial community by affecting the physiological state of cucumber. Furthermore, these results further show that the rhizosphere and bulk microbial communities might respond differently to VA, but this hypothesis should be further tested.

In this study, VA did not alter the cucumber rhizosphere fungal community diversity, as demonstrated by MiSeq sequencing, and this findings do not agree with the results of a PCR-denaturing gradient gel electrophoresis analysis (Zhou and Wu, 2013). Specifically, PCR-DGGE failed to detect some species, possibly due to the presence of several biases, such as the preferential DNA amplification of some DNA templates during PCR, the co-migration of DGGE bands on the gel and the possible presence of faint bands on gel images that might be difficult to detect by the naked eye, leading to an incompletion picture of the microbial diversity (Garofalo et al., 2017; Milanović et al., 2017). However, MiSeq sequencing shows higher efficiency and sensitivity, which makes it possible to detect even taxa found at low abundance (Cardinali et al., 2017). This distinction was also reflected by the higher fugal community richness (OTU number) detected by MiSeq sequencing than the total fugal community richness (number of visible bands) detected by PCR-DGGE. In the present study, the OTU numbers of the *Fusarium* and *Trichoderma* spp. communities detected by MiSeq sequencing were lower than the numbers of visible bands detected by PCR-DGGE. The observed differences might also be due to the different primers used for the PCR protocols in the PCR-DGGE (Yergeau et al., 2005; Meincke et al., 2010) and MiSeq sequencing analyses (Gardes and Bruns, 2010). Thus, different molecular techniques should be combined to characterize the biodiversity of complex ecosystems.

## Conclusion

The data from this study showed that the overall effect of VA, an autotoxin of cucumber, on the composition of the total soil fungal community is limited, however, the application of VA altered the abundances and structures of the *Trichoderma* and *Fusarium* spp. communities. VA also increased the *Trichoderma* and *Fusarium* spp. community abundance at concentrations of 0.05 to 0.2 and 0.02 to 0.05 μmol g^-1^ soil, respectively, and decreased the *Fusarium* spp. community abundance at concentrations from 0.1 to 0.2 μmol g^-1^ soil. VA (0.02 and 0.2 μmol g^-1^ soil) increased the number of bands and the Shannon-Wiener and evenness indices of *Trichoderma* spp., and the application of VA (0.02 and 0.05 μmol g^-1^ soil) increased the band number and the Shannon-Wiener and evenness indices of the *Fusarium* spp. community. Soil microbial communities are responsible for a wide range of soil functions, and ecological changes in microbial communities can influence plant performance (Bever et al., 2012). The changes in soil microbial communities induced by VA might be associated with the adverse effects of VA on cucumber growth under various soil conditions, and this hypothesis should be studied further.

## Author Contributions

XZ and FW conceived and designed the study. SC and HY performed the experiments. SC analyzed the data and wrote the manuscript. All the authors have read and approved the final manuscript.

## Conflict of Interest Statement

The authors declare that the research was conducted in the absence of any commercial or financial relationships that could be construed as a potential conflict of interest.
