# Supplementary material for: Cucumber (Cucumis sativus L.) Seedling Rhizosphere Trichoderma and Fusarium spp. Communities Altered by Vanillic Acid
Source: Front Microbiol. 2018 Sep 18;9:2195. doi: 10.3389/fmicb.2018.02195 (PMC6157394; doi:10.3389/fmicb.2018.02195)
Supplement: Supplementary file 1 [file Data_Sheet_1.doc]

# Cucumber (*Cucumis sativus* L.) seedling rhizosphere *Trichoderma* and *Fusarium* spp. communities altered by vanillic acid

Shaocan Chen ^1^, Hongjie Yu ^1^, Xingang Zhou ^1,2^, Fengzhi WU ^1,2, *^

^1^Department of Horticulture, Northeast Agricultural University, Harbin, China

^2^Key Laboratory of Biology and Genetic Improvement of Horticultural Crops (Northeast Region), Ministry of Agriculture, Harbin, China

**^*^Corresponding author:**
Fengzhi Wu; E-mail address: [fzwu2006@aliyun.com](mailto:fzwu2006@aliyun.com)

Xingang Zhou; E-mail address: 395120625@qq.com

**Figure captions:**

**Figure S1.** α-diversity comparison between cucumber rhizosphere treated with water (W) and vanillic acid (VA). Rarefaction curves (A) and Shannon-Wiener index (B) of soil fungal community based on observed OTUs at 97% similarity.

**Figure S2.** Relative abundances of major fungal phyla, order and family in the vanillic acid and water-treated soils. Fungal phyla with average relative abundances > 1% (A), fungal order with average relative abundances > 7% (B) and > 0. 1% (C), and fungal family with average relative abundances > 5% (E) and > 0. 1% (F) were shown in at least one treatment. Values in the bar plot are expressed as mean ± standard error. The colored circles represent the 95% confidence intervals. Asterisks indicate significant difference between treatments based on Welch’s t test (*P* < 0.05)

**Figure S3.** Diversity and richness indices of soil fungal community in the vanillic acid and water-treated soils. OTUs were delineated at 97% sequence similarity. These indices were calculated using random subsample of 32073 ITS gene sequences per sample. Different letters indicate significant difference based on Welch’s t test (P < 0.05)

Figure S4. PCR-DGGE analysis of the *Trichoderma* and *Fusarium* spp. in cucumber rhizosphere treated with water and vanillic acid. A and B represents DGGE profile of *Trichoderma* and *Fusarium* spp., respectively. 0.02, 0.05, 0.1and 0.2 represent soils amended with vanillic acid at concentrations of 0.02, 0.05, 0.1, 0.2 μmol g^-1^ soil, respectively.

**Table S1.** Accession Number assigned for the different OTUs.

| Treatment | Accession Number |
| --- | --- |
| 0.05-1 | SRR6302352 |
| 0.05-2 | SRR6302353 |
| 0.05-3 | SRR6302354 |
| 0-1 | SRR6302355 |
| 0-2 | SRR6302356 |
| 0-3 | SRR6302357 |

**Figure S1.**


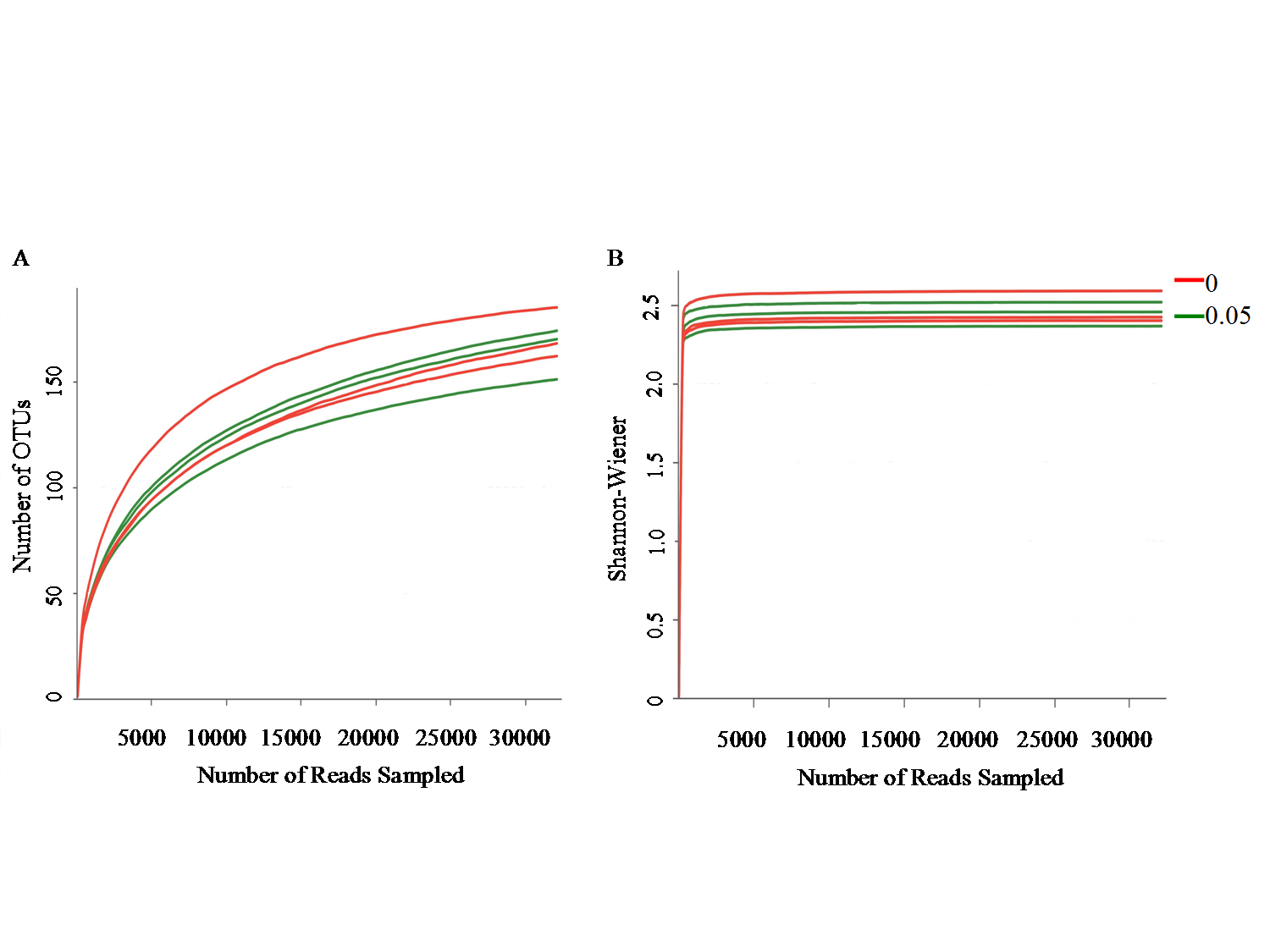


**Figure S2.**


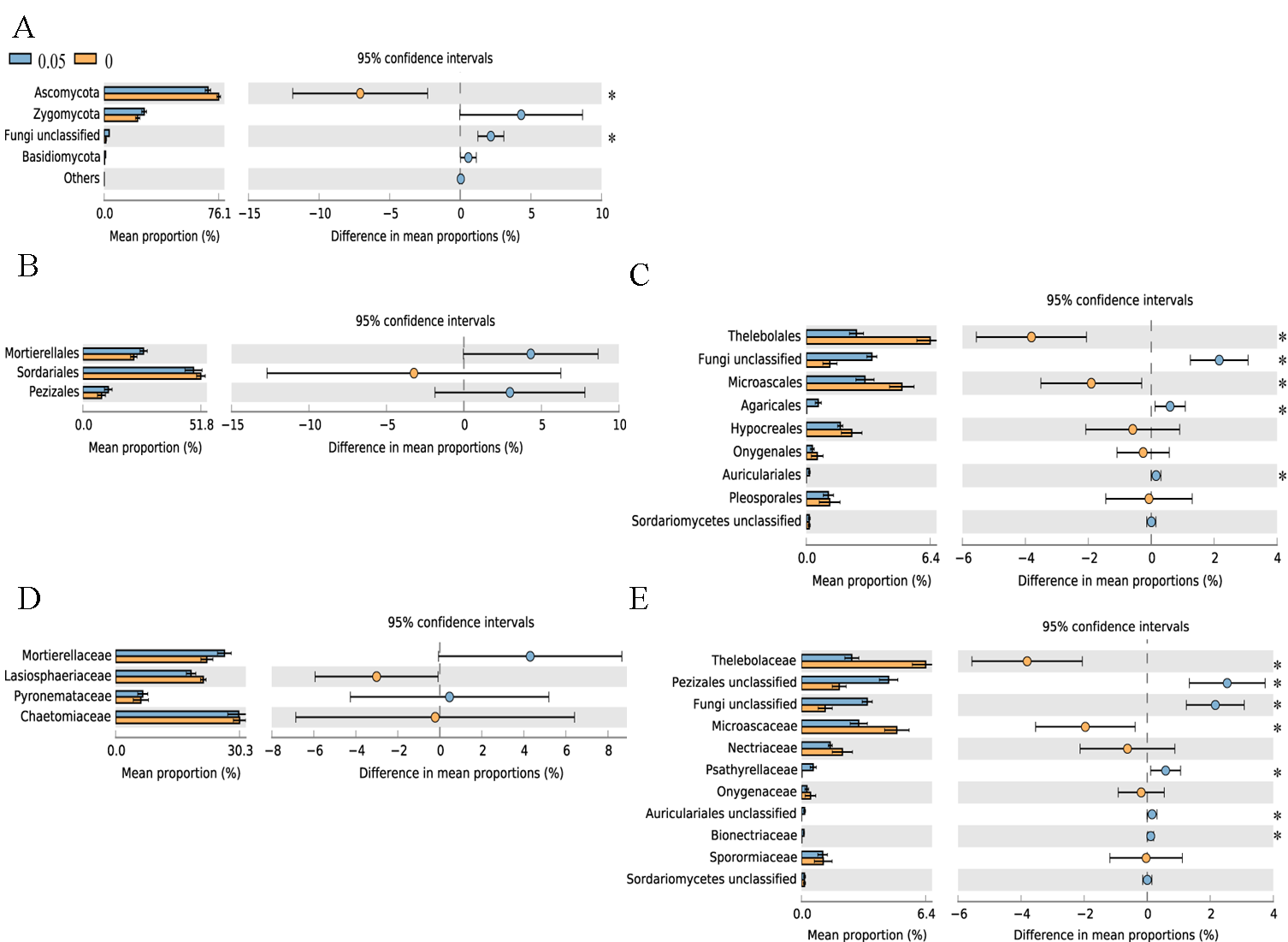


**Figure S3.**


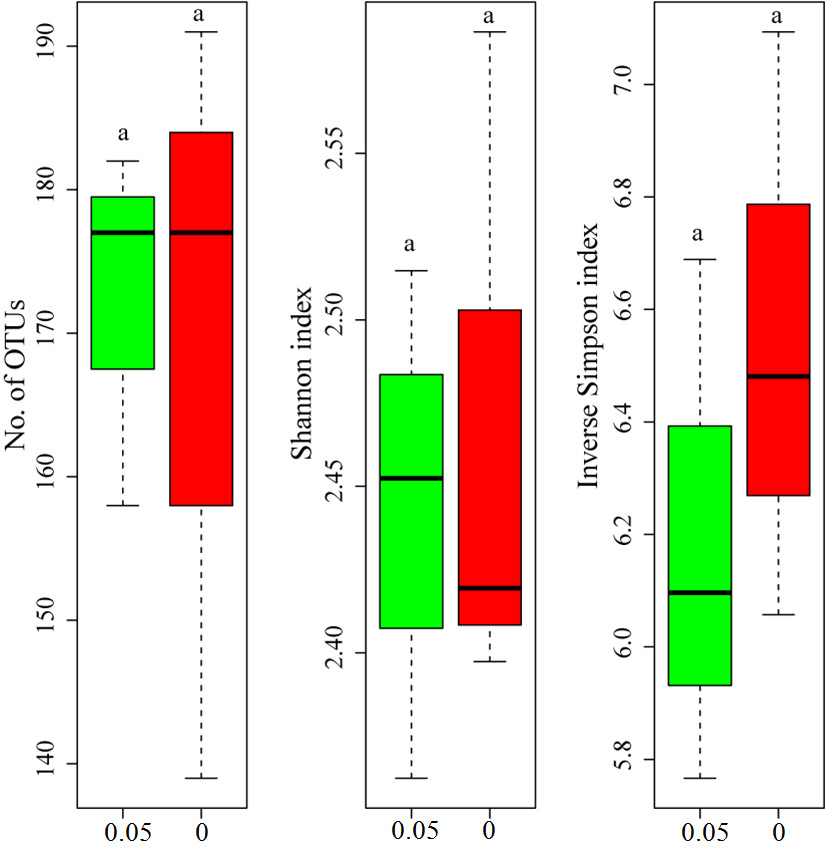


**Figure S4.**

0.2 0.02 0.05 0.1


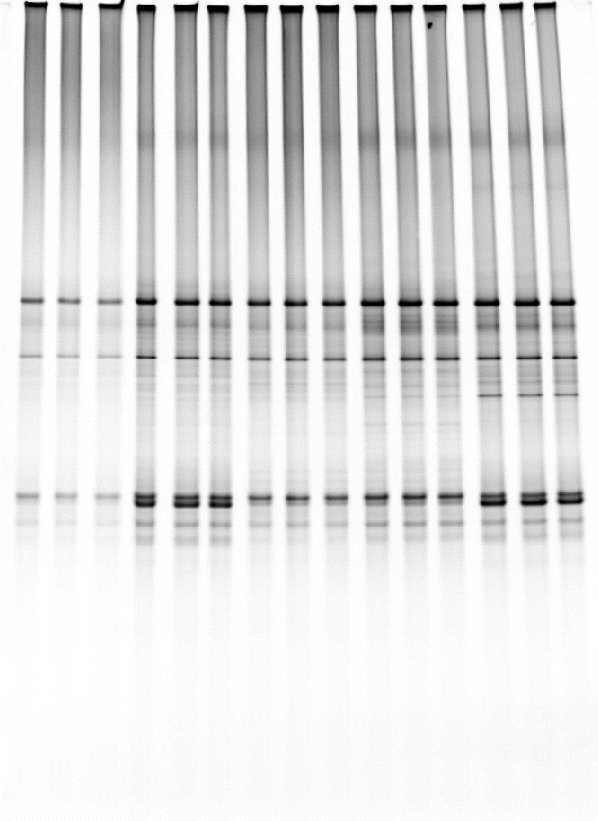


0

B

(a)


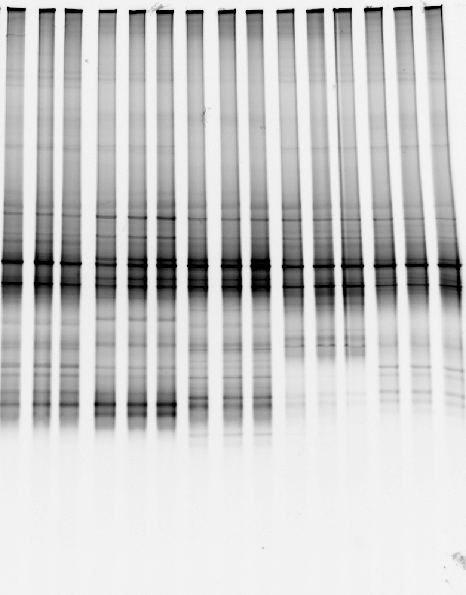


A

0

0.02

0.05

0.1

0.2
